# Influence of Oil–Pressboard Mass Ratio on the Equilibrium Characteristics of Furfural under Oil Replacement Conditions

**DOI:** 10.3390/polym12112760

**Published:** 2020-11-23

**Authors:** Jiefeng Liu, Zhanwei Cao, Xianhao Fan, Heng Zhang, Chuhan Geng, Yiyi Zhang

**Affiliations:** School of Electrical Engineering, Guangxi University, Nanning 530004, China; liujiefeng9999@163.com (J.L.); czw23666@163.com (Z.C.); xianhao_fan@163.com (X.F.); hengzhang_gxu@163.com (H.Z.); 1912301009@st.gxu.edu.cn (C.G.)

**Keywords:** transformer, oil–pressboard insulation, furfural analysis, oil–pressboard mass ratio, oil replacement

## Abstract

The distribution behavior of furfural in insulation systems is influenced by the oil–pressboard mass ratio. In addition, the equilibrium distribution of furfural between oil and pressboard will be disturbed after oil replacement. Therefore, it is of great significance to study the distribution ratio of furfural in oil with various oil–pressboard mass ratios after oil replacement. In this research, an accelerated thermal aging experiment and oil replacement experiment were conducted in the lab. Furthermore, the equilibrium characteristics of furfural dissolved in oil with various oil–pressboard mass ratios were studied. Multiple regression analysis was used to analyze the relationship between the oil–pressboard mass ratios and the distribution ratio of furfural in oil. The equilibrium distribution model of furfural was thus obtained. Afterwards, the modified furfural distribution model under oil replacement conditions was established. A novel scheme is provided for analyzing the equilibrium characteristics of furfural under various oil–pressboard mass ratios after oil replacement. The work of this paper is expected to improve the accuracy of furfural analysis.

## 1. Introduction

Transformers are the core equipment of power transmission in a power grid. The stability and safety of transformers in operation are directly related to the economic benefits of electric utilities [1,2,3,4]. Generally, the main insulation of a power transformer is composed of mineral-insulating oil and insulating cellulose. However, the main insulation of the transformer is irreversibly deteriorated due to the combined effects of electrical, thermal, vibration, and other aging factors during long-term operation [5,6,7,8,9]. Based on the current studies, the aging of cellulose insulation will result in serious failure problems for transformers [10], which may bring security risks to the power system and cause huge socioeconomic losses. Therefore, cellulose insulation determines the service life of the transformer [11,12]. To avoid the occurrence of transformer failure, the aging state of cellulose insulation must be evaluated timely and accurately [13,14,15].

In general, the aging state of the insulating cellulose can be reflected by its tensile strength (*TS*) and degree of polymerization (*DP*) [16,17]. Nevertheless, both methods require destructive sampling of the transformer insulation. As a result, the measurement of dissolved chemicals in the insulating oil is used as an indirect and effective method to evaluate the aging state of cellulose insulation of transformer. Among all the chemical parameters, furan compounds are produced only by the degradation of cellulose insulation [18,19,20,21]. Compared with other furan compounds, furfural is more stable, has a higher content in oil, and is more convenient to detect [22,23]. Therefore, furfural has been widely used as an ideal chemical indicator for characterizing the aging state of cellulose insulation, which has been incorporated into the IEC (International Electrotechnical Commission) 61198.

During the operation of the transformer, part of the furfural molecules diffuses into the insulating oil through the oil–pressboard boundary, which finally achieves equilibrium distribution state in the oil–pressboard system. Consequently, the study of furfural distribution ratio in oil is the premise for evaluating the aging state of cellulose insulation. Reviewing the existing studies, Jalbert et al. studied the effect of temperature on the distribution of furfural between oil and paper, and the results showed that the distribution ratio of furfural in oil increases with rising temperatures [24,25]. Feng et al. studied the influence of moisture on the distribution behavior of furfural, and the results showed that the distribution ratio of furfural in oil is positively correlated with the water content in oil [26]. Moreover, the aging state of insulating paper and oil is another factor that influences the distribution of furfural between oil and paper. Yang et al. found that the distribution ratio of furfural in oil increases with the deterioration of insulating oil [27]. However, the effect of the oil–pressboard mass ratio on furfural distribution behavior has not been well studied. The mass ratio of the oil–pressboard insulation system is different due to the different voltage levels, capacity, and manufacturing design of transformers, which have an effect on the distribution of furfural in oil.

Moreover, the service period of oil-immersed transformers is generally around 40 years. Currently, a large number of field transformers have been in operation for more than 30 years, approaching the end of their service life [28,29]. To slow down the aging rate of the oil–pressboard insulation system, and improve the insulation performance of insulating oil, the transformer oil will be completely replaced by power utilities, which affects the distribution of furfural in oil as well [30].

In this study, the influence of the oil–pressboard mass ratio on the equilibrium distribution characteristics of furfural in oil–pressboard insulation systems was studied. First, ten sample groups were prepared, which included control groups, sample groups, and verification groups with various oil–pressboard mass ratios. Then, the accelerated thermal aging experiment and oil replacement experiment were conducted in the lab. Finally, the distribution model of furfural dissolved in oil was established, and the correction factor of furfural distribution ratio in oil after oil replacement was proposed. A novel scheme is provided for analyzing the equilibrium characteristics of furfural under various oil–pressboard mass ratios after oil replacement.

## 2. Sample Preparation and Experiments

### 2.1. Sample Pretreatment and Accelerated Thermal Aging Experiment

**Figure 1:**
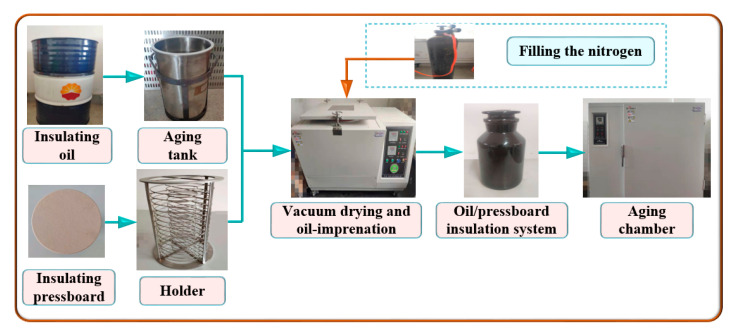
Flow chart of sample pretreatment and accelerated thermal aging experiment.

The oil–pressboard insulation samples used in the experiment were made from no. 25 mineral oil from Karamay Co., Ltd. (Beijing, China), and 1 mm thick cellulose insulation pressboard. The specific experimental material parameters are shown in Table 1. The steps were as follows:

1. First, the insulating pressboard was cut into rectangular pieces (6.0 cm × 3.6 cm) and put on a bracket. Then, the bracket with insulation pressboard and the steel tank containing insulating oil were dried for at least 48 h in the vacuum immersion device at 105 °C for 48 h;
2. Second, the insulation pressboard was immersed in a steel tank containing dry oil in vacuum at 60 °C for 48 h. This ensured that the initial moisture content of the insulating pressboard was less than 0.8%, and that of insulating oil was less than 35 mg/kg;
3. Thirdly, insulating pressboard and oil were placed into ten ground glass bottles, according to different oil–pressboard mass ratios. An appropriate number of copper sheets was put into each bottle to make the thermal aging experiment condition close to the actual operation state of the transformer. Furthermore, each bottle was filled with high-purity nitrogen and strictly sealed. This process was repeated after the oil replacement operation and sampling operation to eliminate the effect of oxygen;
4. Finally, the oil–pressboard insulation samples were put into an aging chamber at 130 °C for an accelerated thermal aging experiment [31,32]. The experimental process is shown in Figure 1.

### 2.2. Oil Replacement Experiment

Ten groups of oil–pressboard insulation samples were set at various oil–pressboard mass ratios. The specific experimental scheme is shown in Table 2. The oil samples of Group A_1_, B_1_, C_1_, D_1_, and E_1_ were regarded as control groups without oil replacement. Group A_2_, B_2_, C_2_, D_2_, and E_2_ were regarded as experimental groups, and the oil replacement experiment was carried out under various oil–pressboard mass ratios.

The accelerated thermal aging experiment lasted for 21 days. On the eighth day, the oil of group A_2_, B_2_, C_2_, D_2_, and E_2_ was completely replaced with fresh oil. Then the five groups of samples were transferred and placed in a thermostat at 60 °C for two weeks until the distribution of furfural between oil and pressboard reached equilibrium state [28].

### 2.3. Sampling and Parameter Measurement

**Figure 2:**
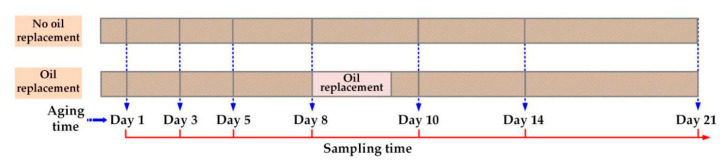
The specific sampling time points.

Samples were taken on days 1, 3, 5, 8, 10, 14, and 21 during the accelerated thermal aging experiment. The specific sampling time points are shown in Figure 2. Before each sampling, each bottle was placed in a thermostat at 60 °C for 72 h to achieve a balanced distribution of furfural between oil and pressboard. In addition, the oil–pressboard mass ratio of each group was maintained constantly throughout the experimental process.

**Figure 3:**
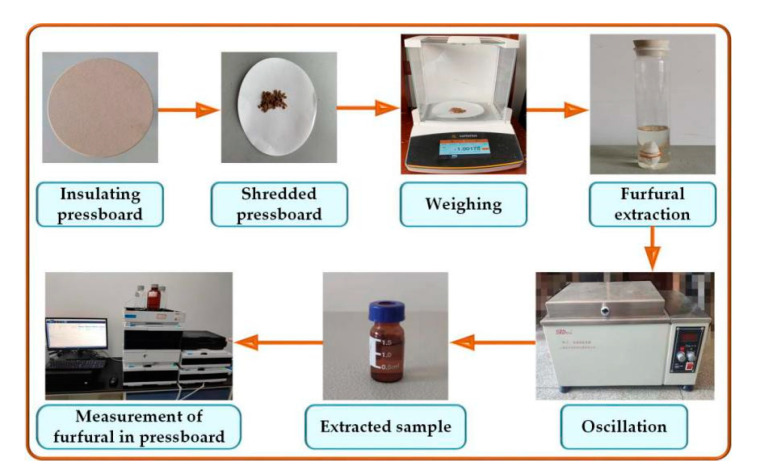
Measurement of furfural concentration in pressboard.

According to IEC 61198, the concentration of furfural dissolved in oil was measured by high-performance liquid chromatography (HPLC). De Pablo extracted furfural from the pressboard with methanol, and the furfural concentration in methanol solvent was measured to obtain the furfural concentration in pressboard [33,34]. As shown in Figure 3, first 1 g of insulating pressboard was taken, cut into small pieces, and put into the colorimetric tube. Secondly, 50 mL methanol was added to the colorimetric tube, and the extraction time lasted for at least 12 h. Thirdly, the concentration of furfural in methanol was measured by HPLC, and the furfural concentration in insulating pressboard was calculated by Equation (1). According to IEC 60450, insulating pressboard was dissolved in copper ethylenediamine solution, and the degree of polymerization of the insulating pressboard was measured by viscosity method [17].

(1) $$C_{pressboard}=\frac{C_{meoh}\times V_{meoh}}{m_{pressboard}}$$

where *C_pressboard_* (mg/g) is the concentration of furfural in insulating pressboard, *C_meoh_* (mg/L) is the concentration of furfural in methanol, *V_meoh_* (L) is the volume of methanol, and *m_pressboard_* (mg) is the mass of insulating pressboard.

## 3. Experimental Results

### 3.1. Relationship Between Aging Time and Furfural Concentration

**Figure 4:**
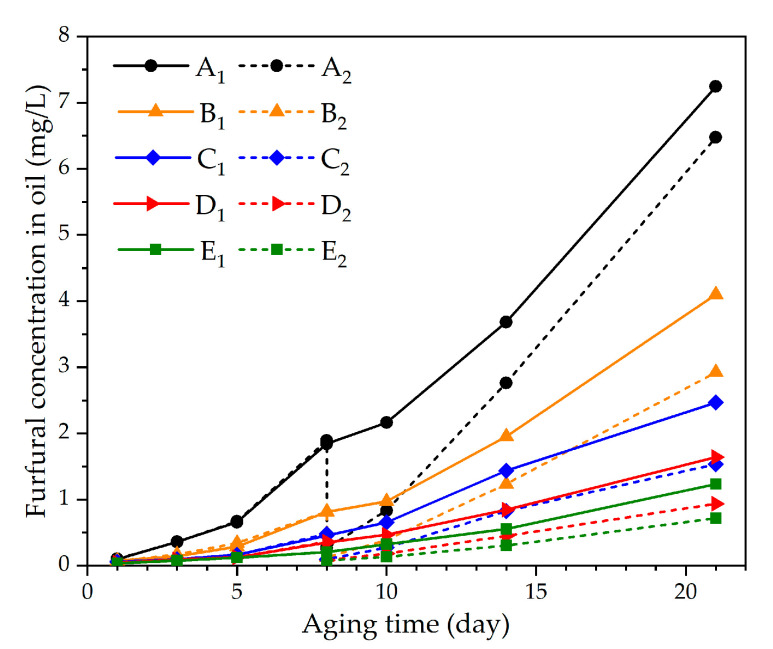
The relationship between furfural concentration in oil and aging time.

**Figure 5:**
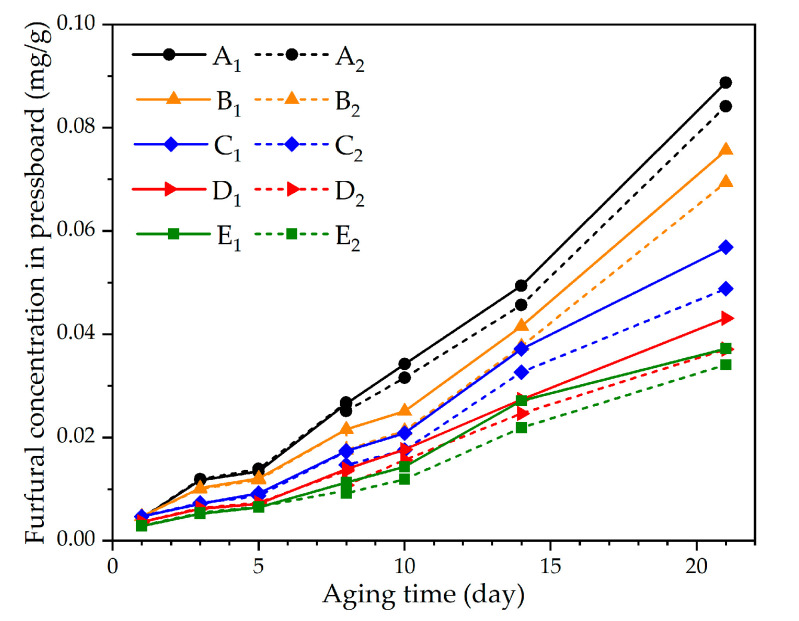
The relationship between furfural concentration in pressboard and aging time.

The furfural concentration in the oil and pressboard increased with the aging of the sample in the control groups (A_1_, B_1_, C_1_, D_1_, and E_1_). The oil in experimental groups A_2_, B_2_, C_2_, D_2_, and E_2_ was totally replaced under various oil–pressboard mass ratios on the eighth day of the accelerated thermal aging experiment (*DP* is about 530). Those oil–pressboard mass ratios were maintained at 5:1, 10:1, 15:1, 20:1, and 25:1, respectively. The concentrations of furfural in oil and pressboard were measured on days 1, 3, 5, 8, 10, 14, and 21 of the accelerated thermal aging experiment. The results are shown in Figure 4 and Figure 5.

As shown in Figure 4 and Figure 5, the furfural concentration in the oil and pressboard decreased with the increase of the oil–pressboard ratio. Although the furfural content in the oil decreased significantly after the oil replacement, the furfural content did not decrease to 0. It is indicated that the insulating oil could not be completely removed by oil replacement, and a small part of the insulating oil remained on the surface of the insulating pressboard and the inner wall of the bottle. The furfural residual ratio in oil increased with the increase of the oil–pressboard mass ratio, as illustrated in the Table 3, while the furfural recovery ratio in oil decreased with the increase of the oil–pressboard mass ratio.

As shown in Figure 4, the higher the oil–pressure mass ratio, the greater the furfural loss. After oil replacement, the accelerated thermal aging experiment was carried out. The furfural concentration in the group with a lower oil–pressboard mass ratio increased faster than that of the group with the higher oil–pressboard mass ratio. In group E_2_, after 21 days of accelerated thermal aging (*DP* was about 300), the concentration of furfural dissolved in oil was only 58.1% of that in the group without oil replacement (Group E_1_). Also, the furfural concentration in the oil at the end stage of aging decreased when the oil–pressboard mass rate increased.

The polar molecules, such as water and acid molecules, in the oil were greatly decreased after oil replacement, and the polarity of the insulating oil was also greatly decreased. Consequently, the absorption capacity of insulating oil to furfural molecules was decreased. As a result, polar molecules were hardly diffused into the oil through the oil–pressboard boundary [35].

### 3.2. Furfural Concentration in the Oil and Pressboard

**Figure 6:**
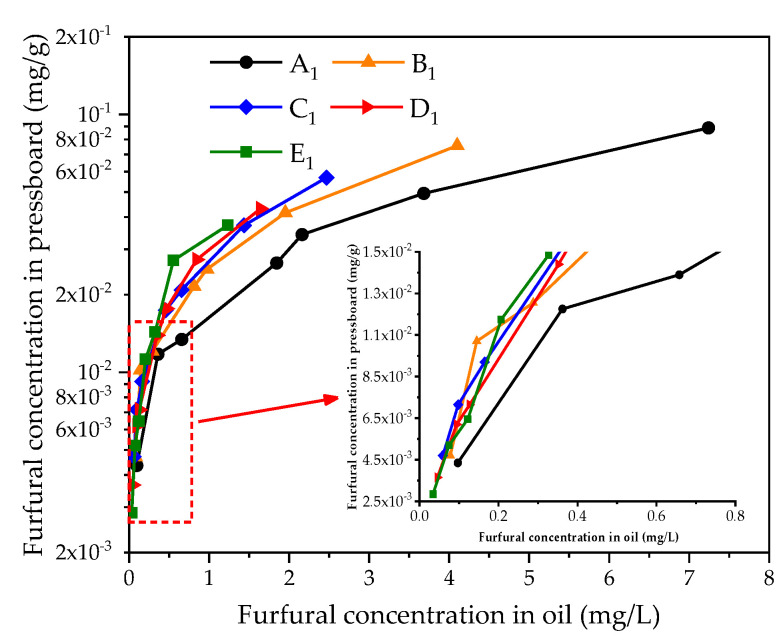
The relationship between furfural concentration in oil and that in pressboard.

As shown in Figure 6, the furfural concentration in the oil increases with the increase of the furfural concentration in the pressboard. The lower the oil–pressboard mass ratio is, the higher the furfural concentration in the oil and pressboard, and the faster the furfural concentration increases.

### 3.3. The DP of Pressboard and Furfural Concentration in Oil

**Figure 7:**
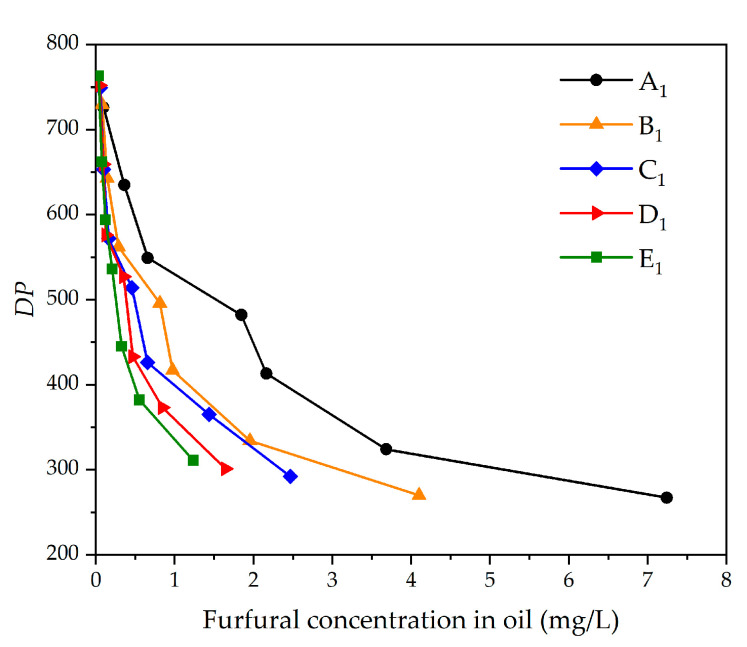
The relationship between furfural concentration oil and degree of polymerization (*DP*).

As shown in Figure 7, the furfural concentration in oil increases with the decrease of *DP*. At the same aging degree, the furfural concentration in oil and pressboard increases as the mass ratio of oil to pressboard decreases. At the same time, the growth rate of furfural concentration is inversely proportional to the decrease of the oil–pressboard mass ratio.

## 4. Data Analysis and Model Building

### 4.1. Equilibrium Distribution Ratio of Furfural

**Figure 8:**
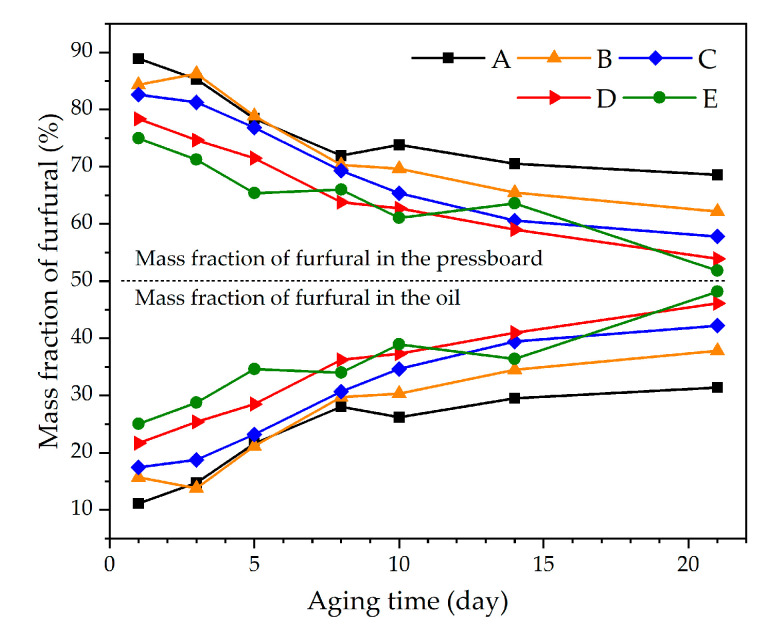
The relationship between furfural mass fraction and aging time.

**Figure 9:**
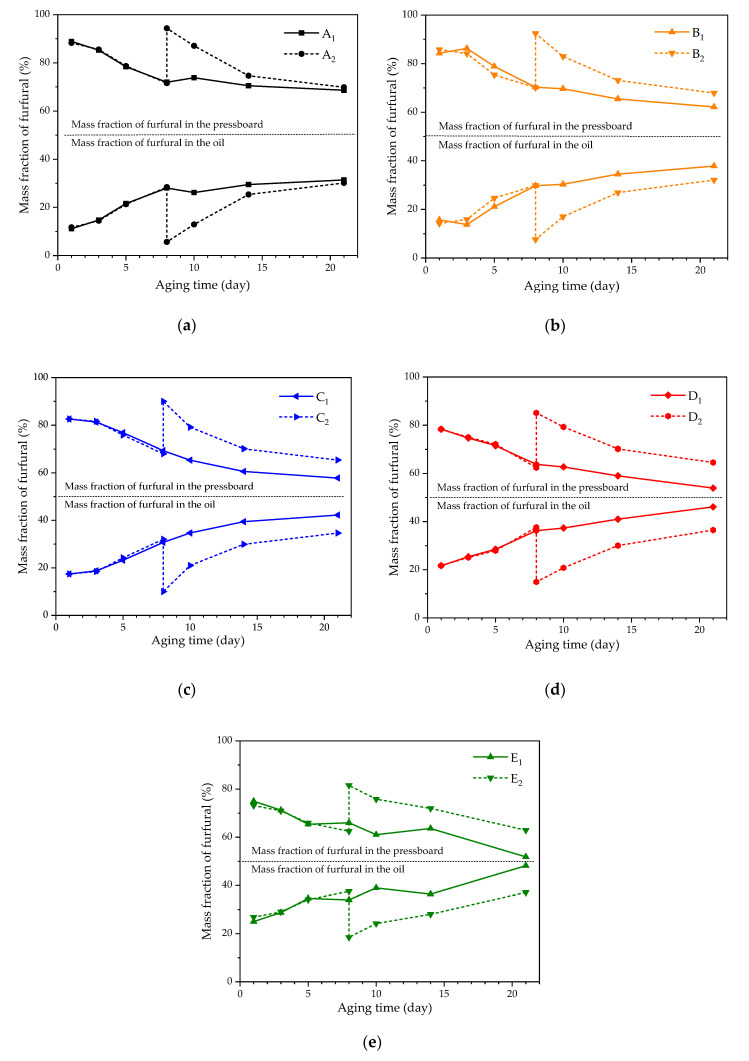
The relationship between furfural mass fraction and aging time after oil replacement. (**a**) Effects of oil replacement on the partitioning of furfural in group A. (**b**) Effects of oil replacement on the partitioning of furfural in group B. (**c**) Effects of oil replacement on the partitioning of furfural in group C. (**d**) Effects of oil replacement on the partitioning of furfural in group D. (**e**) Effects of oil replacement on the partitioning of furfural in group E.

Data from groups A_1_, B_1_, C_1_, D_1_, and E_1_ were used to analyze the influence of aging degree and oil–pressboard mass ratio on furfural distribution ratio. The furfural mass fraction was used to characterize the equilibrium distribution ratio of furfural in oil–pressboard insulation systems, as shown in Equations (2) and (3). The experimental results are shown in Figure 8 and Figure 9.

(2) $$w_{oil}\;=\frac{\;Coil\times Voil}{Coil\times Voil+Cpressboard\times Mpressboard}$$

(3) $$w_{pressboard}\;=\frac{\;Cpressboard\times Mpressboard}{Coil\times Voil+Cpressboard\times Mpressboard}$$

where *C_oil_* is the concentration of furfural in insulating oil, *V_oil_* is the volume of insulating oil *w_oil_ i*s the mass fraction of furfural in the oil and *w_pressboard_* is the mass fraction of furfural in the pressboard.

It can be concluded from Figure 8 that the mass fraction of furfural in oil increased with aging time, while the mass fraction of furfural in the pressboard decreased. It can be easily found that the degree of aging has a significant influence on the distribution ratio of furfural in the oil–pressboard system.

After oil replacement, it can be concluded from Figure 9 that, the distribution ratio of furfural in oil decreased with the increase of oil–pressboard mass ratio. Most of the small molecule polar substances in insulating oil were lost due to oil replacement. Furthermore, the low concentration of small molecular polar substances in the new oil had weak adsorption capacity to furfural. After oil replacement, the proportion of furfural in oil increased with the deterioration of insulating oil. With the further deepening of the aging degree, the number of polar substances in insulating oil increased significantly, which enhanced the adsorption capacity of oil to furfural. However, after 21 days of accelerated thermal aging (*DP* was about 300), the distribution ratio of furfural in oil after oil replacement was lower than that without oil replacement. The results showed that oil replacement had a significant effect on the distribution ratio of furfural in the oil–pressboard system.

**Figure 10:**
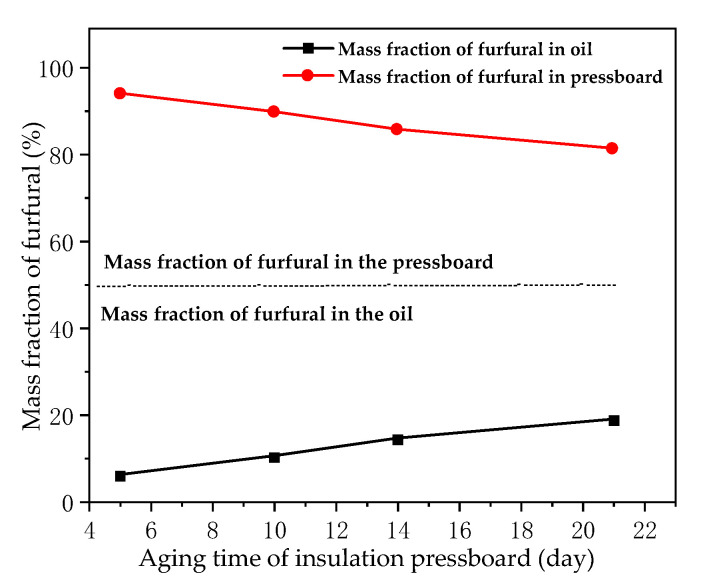
Mass fraction of furfural in groups composed of new oil and pressboard with different aging degrees.

**Figure 11:**
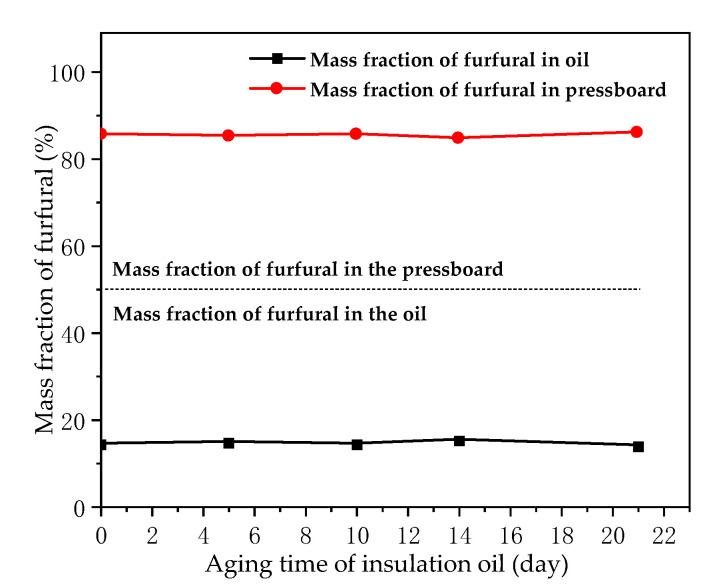
Mass fraction of furfural in groups composed of aged pressboard and oil with different aging degrees.

The effects of aging degree of oil and pressboard on furfural distribution behavior in oil were studied. A total of eight sample groups (Rs) were used. R_1_ to R_4_, which were used to investigate the effect of different aging degrees of insulating pressboard on the distribution of furfural between oil and pressboard under new oil environments. R_3_ and R_5_–R_8_ were used to explore the effect of different aging degrees of insulating oil on the distribution of furfural between oil and pressboard with the same aging degree of pressboard, as shown in Table 4. The experimental data are shown in Figure 10 and Figure 11.

The experimental results indicated in Figure 10 show that, under the premise of the same aging degree of the insulating oil, the deeper the aging degree of pressboard, the greater the mass fraction of furfural in the oil. Therefore, the aging degree of the insulating pressboard determines the distribution of furfural in the oil–pressboard insulation system. Figure 11 shows that, under the premise of the same aging degree of the insulating pressboard, the aging degree of the insulating oil has little effect on the mass fraction of furfural in the oil–pressboard system. Therefore, the aging degree of the insulating oil does not affect the equilibrium distribution of furfural in the oil–pressboard insulation system. Combined with the results of Figure 10 and Figure 11, it can be concluded that the aging degree of pressboard is the main factor affecting the equilibrium distribution of furfural between the oil and pressboard. The aging of the insulating pressboard will promote the diffusion of furfural from the insulating pressboard to the insulating oil.

### 4.2. The Establishment of the Equilibrium Distribution Model

The model of furfural equilibrium distribution between oil and pressboard was established and validated in 35 data sets of oil–pressboard insulating samples, with seven types of oil–pressboard aging degrees and five types of oil–pressboard mass ratios. Thirty groups of data were selected from 35 groups to establish the equilibrium distribution model. The distribution ratio of furfural dissolved in oil was taken as the dependent variable, while oil–pressboard mass ratio and degree of polymerization of the insulating pressboard were taken as independent variables. The equilibrium distribution model of furfural established is shown in Equation (4).

(4) $$w_{oil}\;=\;a+b\times MR+c\times DP$$

**Figure 12:**
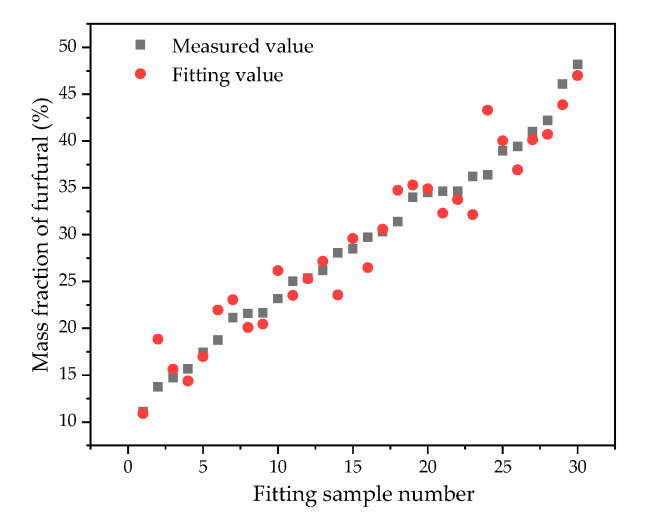
The fitting results of the model of furfural equilibrium distribution.

In Equation (4), *w*_oil_ represents the furfural distribution ratio in oil (%), *MR* represents the oil–pressboard mass ratio, and *DP* represents the degree of polymerization of insulating pressboard. In addition, *a* = 0.450, *b* = 0.0073, and *c* = −0.00052. The fitting results of 30 groups of samples are shown in Figure 12. The goodness of fit *R*^2^ = 0.928 indicates that the model is in good agreement with the actual value. As shown in Table 5, *t*-values were used to determine significance, and when the significance level is less than 0.05, it indicates that the regression equation is effective.

**Figure 13:**
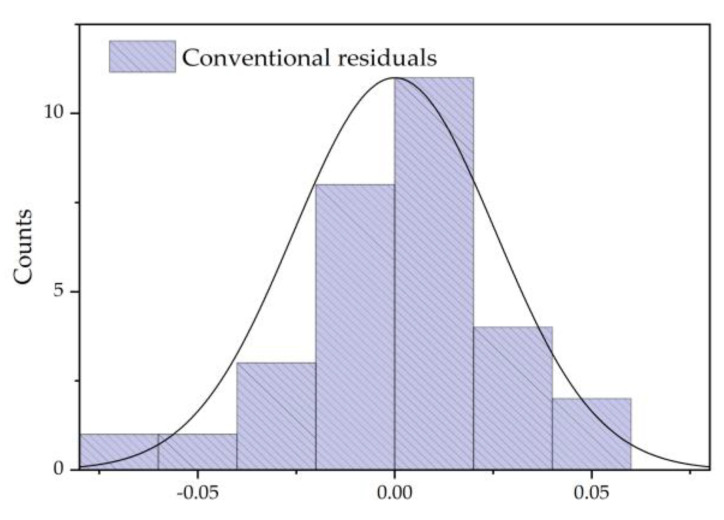
Conventional residual analysis.

As shown in Figure 13, the residual error is fitted. The results show that the residual is in good agreement with the normal distribution, and the mean value was zero. Therefore, the equilibrium distribution model of furfural between oil and pressboard is effective.

The distribution model of furfural in oil–pressboard was verified by five groups of data from 35 groups of samples. Table 6 shows the oil–pressboard mass ratio (*MR*), degree of polymerization (*DP*), the actual distribution ratio of furfural in oil obtained in the experiment (*w*_oil_), and the predicted value calculated by five verification samples, according to Equation (4). The accuracy (%) is the ratio of the calculated value to the actual value.

**Figure 14:**
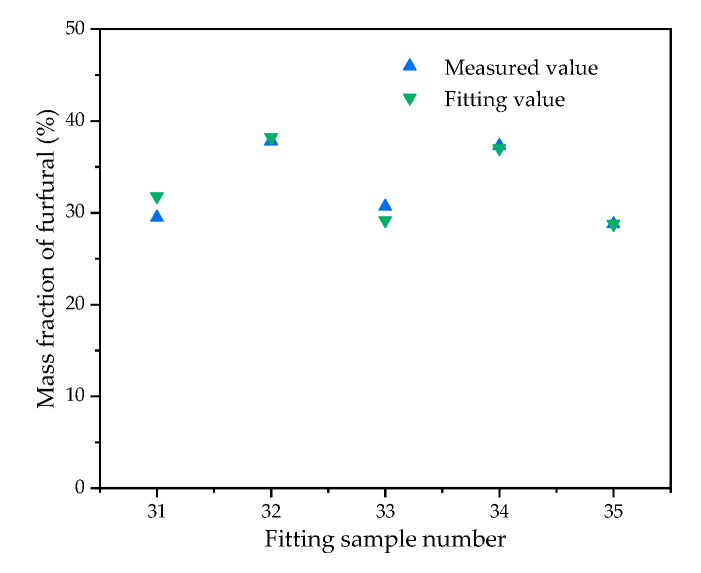
Comparison between the predicted value and measured value of furfural distribution ratio in oil.

It can be seen from Table 6 that the furfural distribution model between oil and pressboard can accurately predict the distribution ratio of furfural dissolved in oil with various oil–pressboard mass ratios and aging degrees, so the equilibrium distribution model is effective. Figure 14 shows the comparison between the predicted value and measured value of furfural distribution ratio in oil.

### 4.3. The Influence of Oil Replacement on the Equilibrium Distribution Ratio of Furfural and Correction

Data from groups A_2_, B_2_, C_2_, D_2_, and E_2_ were used to analyze the influence of oil replacement on the furfural distribution ratio. The furfural concentration in oil was greatly lost after oil replacement, and the proportion of furfural in oil decreased dramatically.

The expression of the correction factor for the equilibrium distribution ratio of furfural in oil is shown in Equation (5), where *t* is the aging time, *t*_re_ is the time point of oil replacement, *w*_0_(*t*) represents the distribution ratio of furfural in oil at time *t* without oil replacement, and *w*_1_(*t*) is the distribution ratio of furfural in oil at time *t* after oil replacement.

(5) $$f\left(t\right)\;=\;\{\begin{array}{cc} 1 & t<tre\\ \frac{w_{1}\left(t\right)}{w_{0}\left(t\right)} & t\geq tre \end{array}$$

In both Equations (4) and (5), the modified furfural distribution model under oil replacement conditions is established, as shown in Equation (6), where the parameter *w_c_* represents the distribution ratio of furfural in oil after the correction of the oil replacement sample.

(6) $$w_{c}=\frac{w_{1}\left(t\right)}{f\left(t\right)}=\;0.450+0.0073\times MR-0.00052\times DP$$

**Figure 15:**
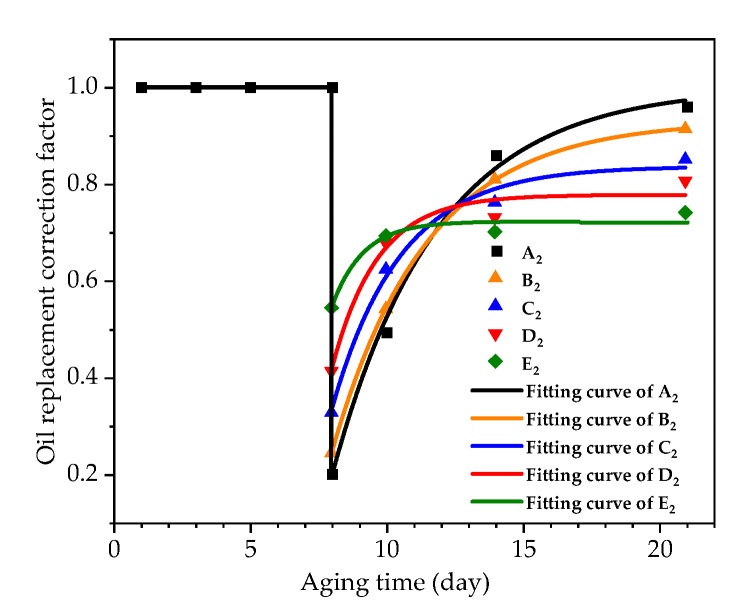
The relationship between oil replacement correction factor and aging time after oil replacement.

According to the experimental results, the relationship between the oil replacement correction factor for the furfural distribution ratio in oil and aging time was obtained by Equation (6), as shown in Figure 15. After the oil replacement, the correction factor suddenly dropped to around 0.2. At the initial stage after the oil replacement, the furfural distribution ratio correction factor in oil increased rapidly, and then the rate of increase slowed down over time. Equation (7), in which *t* is the aging time and *t_re_* is the time point of oil replacement, was used to fit the oil replacement correction factor. The fitting results of each constant are shown in Table 7.

(7) $$f\left(t\right)\;=\;\{\begin{array}{cc} 1 & t<tre\\ m+n\times e^{-k\left(t-t_{re}\right)} & t\geq tre \end{array}$$

The parameter *m* represents the correction factor for the equilibrium distribution ratio of furfural in oil as time goes to infinity (*t* = ∞) after the oil replacement. The parameter *n* is preexponential factor, parameters *m* + *n* represents the correction factor for the equilibrium distribution ratio of furfural in the oil at the moment of oil replacement. The parameter *k* represents the increasing speed of the furfural distribution ratio correction factor, which increases with the increase of oil–pressboard mass ratio. *R*^2^ stands for the goodness of fit of the parameters.

**Figure 16:**
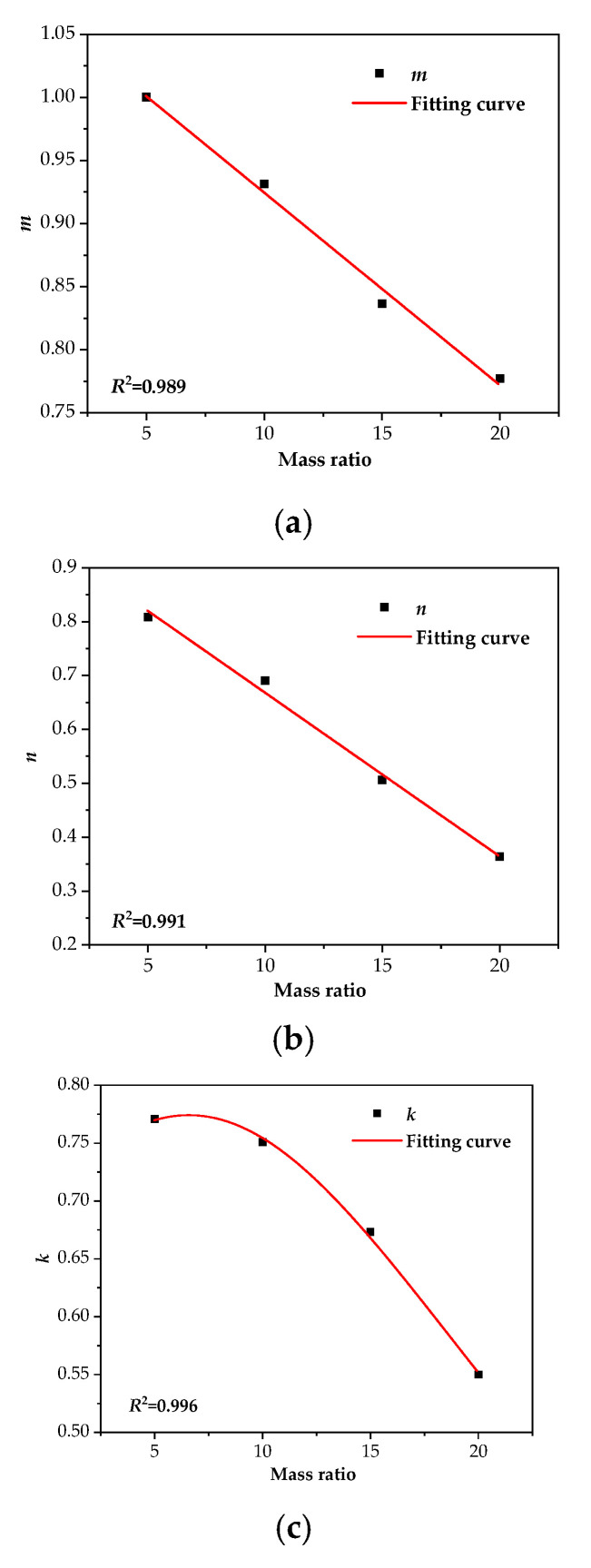
The relationship between oil–pressboard mass ratio (*MR*) and parameters *m*, *n*, and *k*. (**a**) The relationship between *m* and *MR*. (**b**) The relationship between *n* and *MR*. (**c**) The relationship between *k* and *MR*.

Based on the results of groups A2, B2, C2, and D2 from Table 7, the functional relationship between *m*, *n*, *k*, and the oil replacement ratio were established respectively, as shown in Equation (8) and Figure 16.

The parameter *MR* represents the oil–pressboard mass ratio.

(8) $$\{\begin{array}{l} m\;=\;-0.0153\times MR+1.077\\ n\;=\;0.03\times MR-0.972\\ k\;=\;\frac{1}{1.418-0.0383\times MR+0.00289\times MR^{2}} \end{array}$$

**Figure 17:**
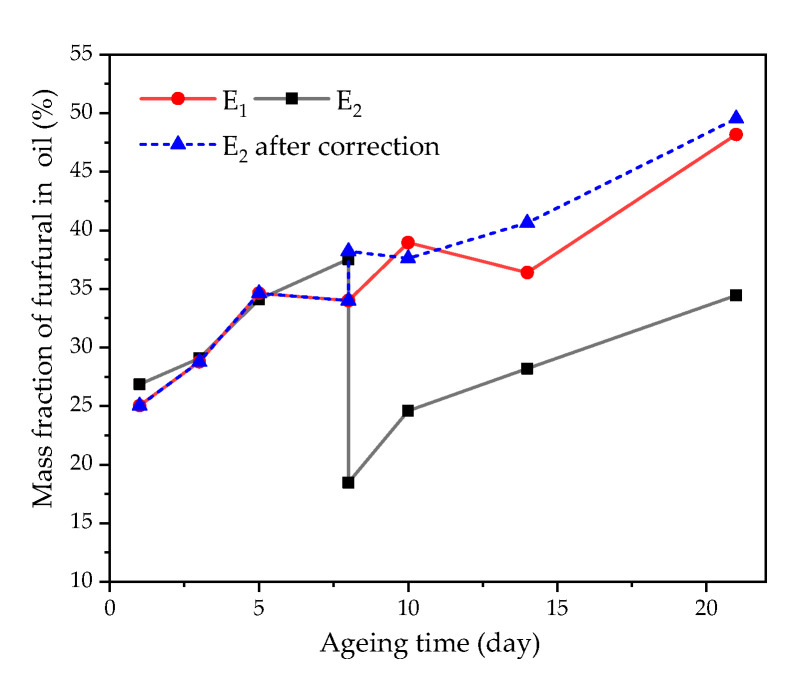
The relationship between furfural mass fraction and aging time after oil replacement.

The oil–pressboard mass ratio of group E_2_ (*ε* = 25) is taken into Equation (8) to calculate the corresponding *m*, *n*, and *k* values, and then *m*, *n*, and *k* are brought into Equation (7) to obtain the furfural distribution ratio correction factor of group E_2_. After introducing the correction factor of the furfural distribution ratio in oil into Equation (6), the distribution ratio deviation caused by oil replacement was well-corrected, and the corrected results were close to the real value, as shown in Figure 17, which proved the feasibility of the proposed model.

## 5. Conclusions

In this paper, the effect of oil replacement on the distribution behavior of furfural in oil under different oil–pressboard mass ratio systems was investigated. After accelerated thermal aging experiments and furfural equilibrium experiments, the results show that the oil–pressboard mass ratio will obviously affect the content and distribution of furfural in oil. The equilibrium distribution model of furfural under different oil–pressboard mass ratios was established, and the fitting data were verified by multiple linear regression. The correction model of the equilibrium distribution of furfural after oil replacement was obtained and verified by the validation group data. The main conclusions are as follows:

1. Furfural partitioning between oil and pressboard was influenced by the oil–pressboard mass ratio. The mass fraction of the furfural in oil increased with the decrease of the oil–pressboard mass ratio. In addition, the experiment shows that the aging degree of pressboard is an important factor that determines the distribution behavior of furfural between oil and pressboard, while the aging degree of oil has little effect on the distribution behavior of furfural;
2. The equilibrium distribution model of furfural was then established. The results show that the proportion of furfural in oil is also affected by the aging degree of insulating pressboard: the deeper the age of the insulating pressboard, the greater the distribution ratio of furfural in the oil;
3. The modified model of furfural distribution ratios in oil after oil replacement was established. The lower the oil–pressboard mass ratio is, the less oil replacement operations influence the furfural distribution in oil. This paper provides a new reference for the equilibrium distribution of furfural in insulation systems, which is expected to improve the accuracy of transformer aging state assessment based on furfural analysis in oil.

This paper investigates the validation of the equilibrium distribution model of furfural based on laboratory data, thus providing a theoretical basis for furfural distribution in field transformers. However, there is difficulty in sampling field transformer paper, which can cause damage to the insulation; in the absence of data on furfural in insulating paper, the equilibrium distribution model of furfural cannot be validated by the field data. Therefore, we will collect data from some field transformers to verify the furfural equilibrium distribution model in further research.

## Figures and Tables

**Table 1:** Parameters of the materials.

Cellulose Pressboard		Insulation Oil		Methanol	
Brand	Weidmann T_4_ pressboard	Brand	Karamay no. 25 naphthenic mineral oil	Brand	Fisher
Provider	Taizhou Weidmann high voltage insulation Co. LTD, Taizhou, China	Provider	China National Petroleum Corporation. Beijing, China	Provider	Thermo Fisher Scientific. Ottawa, Canada
Diameter	160 mm	Pourpoint	−45 °C	Purity	99.9%
Thickness	1 mm	Flashpoint	135 °C		
Tensile strength	MD ^a^ 105 MPa CMD ^b^ 80 MPa	Dielectric loss	4 × 10^−4^	Density	0.791 g/ml

^a^ MD: Machine Direction. ^b^ CMD: Cross-Machine Direction.

**Table 2:** Experimental scheme.

Group	Oil Replacement Ratio	Oil–Pressboard Mass Ratio
A_1_	0%	5:1
A_2_	100%	5:1
B_1_	0%	10:1
B_2_	100%	10:1
C_1_	0%	15:1
C_2_	100%	15:1
D_1_	0%	20:1
D_2_	100%	20:1
E_1_	0%	25:1
E_2_	100%	25:1

**Table 3:** Furfural residual and recovery ratio.

Group	Oil–Pressboard Mass Ratio	Oil Replacement Ratio	Furfural Residual Ratio	Furfural Recovery Ratio
A_2_	5:1	100%	14.2%	89.4%
B_2_	10:1	100%	14.8%	71.3%
C_2_	15:1	100%	20.1%	62.2%
D_2_	20:1	100%	23.1%	65.6%
E_2_	25:1	100%	35.7%	51.7%

**Table 4:** Formation of the eight special sample groups.

Sample Group	Sample Detail	Oil–Pressboard Mass Ratio
R_1_	Pressboard aged for 5 days + new oil	10:1
R_2_	Pressboard aged for 10 days + new oil	10:1
R_3_	Pressboard aged for 14 days + new oil	10:1
R_4_	Pressboard aged for 21 days + new oil	10:1
R_5_	Pressboard aged for 14 days + oil aged for 5 days	10:1
R_6_	Pressboard aged for 14 days + oil aged for 10 days	10:1
R_7_	Pressboard aged for 14 days + oil aged for 14 days	10:1
R_8_	Pressboard aged for 14 days + oil aged for 21 days	10:1

**Table 5:** The goodness of fit of furfural equilibrium distribution model.

Parameters	Standard Error	*t*-Value	Significance Level
*MR*	6.86 × 10^−4^	10.59	<0.05
*DP*	3.23 × 10^−5^	−16.06	<0.05

**Table 6:** Calculation results of the validation groups.

Group	*MR*	*DP*	*W*_oil_ (%)	Predicted Value (%)	Accuracy (%)
S_31_	5	324	29.5	31.8	92.23
S_32_	10	270	37.8	38.2	98.94
S_33_	15	514	30.7	29.2	95.08
S_34_	20	433	37.3	37.0	99.26
S_35_	25	662	28.8	28.8	99.99

**Table 7:** The fitting results of each constant.

Group	*m*	*n*	*k*	*R* ^2^
A_2_	1.000	−0.809	0.26	0.985
B_2_	0.931	−0.690	0.287	0.999
C_2_	0.836	−0.506	0.396	0.975
D_2_	0.777	−0.364	0.598	0.922
E_2_	0.722	−0.179	0.868	0.899
